# Biological Therapies of Severe Asthma and Their Possible Effects on Airway Remodeling

**DOI:** 10.3389/fimmu.2020.01134

**Published:** 2020-06-18

**Authors:** Grzegorz Kardas, Piotr Kuna, Michał Panek

**Affiliations:** Clinic of Internal Medicine, Asthma and Allergy, Medical University of Lodz, Łódz, Poland

**Keywords:** asthma, airway remodeling, airway remodeling in asthma, biological therapy, omalizumab, mepolizumab/reslizumab, benralizumab

## Abstract

Asthma is a chronic and heterogenic respiratory tract disorder with a high global prevalence. The underlying chronic inflammatory process and airway remodeling (AR) contribute to the symptomatology of the disease. The most severely ill asthma patients may now be treated using a variety of monoclonal antibodies aiming key inflammatory cytokines involved in asthma pathogenesis. Although clinical data shows much beneficial effects of biological therapies in terms of reduction of exacerbation rates, improvement of lung functions, asthma control and patients' quality of life, little is known on the effects of these monoclonal antibodies on AR—a key clinical trait of long-term asthma management. In this review, the authors summarize the data on the proven effects of monoclonal antibodies in asthma on AR. To date, in terms of reversing AR, the mostly studied was omalizumab. However, some studies also addressed this clinical issue in context of other severe asthma biological therapies (mepolizumab, benralizumab, tralokinumab). Still, data on effects of particular biological therapies on AR in severe asthma are incomplete and require further studies. According to the American Thoracic Society research recommendations, future research shall focus on AR in asthma and improve drugs targeting AR, including the available and future monoclonal antibodies.

## Introduction

Asthma is a chronic, heterogeneous and inflammatory respiratory condition characterized by shortness of breath, cough, wheezing, and chest tightness. It belongs to the group of obstructive diseases for which the variable airflow limitation is characteristic (1). Various asthma phenotypes differ in causes and mechanism of symptom formation, and thus in severity and frequency of symptoms and exacerbations (2). Currently, asthma affects 1–18% of the population in various countries (1, 3, 4). It occurs in all age groups, with new diagnoses mostly made in children aged 0–9 [early-onset asthma, usually atopic (5)] and in adults aged 40–49 [late-onset asthma, often with eosinophilic phenotype (6)]. Noteworthy is that not only asthma, but also other allergic diseases'–urticaria, allergic rhinitis or food allergies—prevalence increase worldwide (4, 7, 8). The causes of this epidemiological phenomenon mainly include: environmental changes associated with the modification of the surrounding microbiome affecting microbiological and immunological changes in the human respiratory tract from the earliest years of life (the so-called “hygiene theory”), past respiratory infections, exposure to allergens, air pollution, and other pollutants (9, 10).

Currently, “asthma” is considered an umbrella term, which encompasses several, both clinically and pathophysiologicaly different variants of the disease. The two main divisions concern the type of inflammation: T_h_2-predominant and non-T_h_2. Further, phenotypes are distinguished as either eosinophilic asthma or non-eosinophilic (11). In particular, among T_h_2-predominant phenotypes, the most prevalent endotype is the allergic asthma. It develops on basis of atopy, in particular in response to inhaled allergens such as house dust mites, grass pollen, trees, and pets (6). Apart from classical childhood-onset allergic asthma, late-onset eosinophilic asthma is now one of the best-defined phenotypes (12). Several other endotypes of asthma include obesity-associated asthma, neutrophilic asthma, very-late onset asthma and other.

In the pathogenesis of the disease, mediators of the T_h_2-dependent reaction play a key role, including: IgE, IL-3, IL-4, IL-5, IL-13, IL-33, TSLP, and other (13). In non-allergic asthma, although the cellular pathomechanism is different, most of the mediators remain the same, with main variations including IL-17 and PGD_2_. The underlying immunopathological mechanisms of asthma lead to chronic airway inflammation resulting in number of consequences for the bronchi. The airways become hypersensitive and constrict when subject to stressful stimuli. Another result of this ongoing inflammation is airway remodeling (AR), a process of structural changes of bronchi walls. The chronic airway inflammation thus leads to reduced airway airflow and clinical symptoms—wheezing, shortness of breath cough, chest tightness. Unfortunately, those symptoms are few and non-specific, thus the differential diagnosis of asthma is often difficult. Additionally, asthma is often associated with comorbidities, including: other allergic conditions (rhinosinositus, nasal polyps, atopic dermatitis), obesity, diabetes, gastroesophageal reflux, depressive and anxiety disorders, and other (14).

In clinical practice, we distinguish 3 levels of asthma severity (mild, moderate and severe) and 5 Global Initiative for Asthma (GINA) treatment steps (1). Mild asthma comprises of GINA steps 1. and 2. Moderate asthma, characterized by more severe symptoms and more frequent exacerbations is GINA step 3. Severe asthma are GINA steps 4. and 5. The most severely ill patients, i.e., those who do not achieve asthma control despite using high doses of inhaled steroids, are qualified for step 5. biological treatment with monoclonal antibodies against key asthma mediators. The overall clinical goal in asthma is disease control, i.e., a therapy that provides optimal symptom reduction. Drugs and their doses are modified depending on the symptoms, severity of the disease and exacerbation frequency. Treatment may be intensified in absence of control or reduced if long-term, optimal disease control is achieved.

## Airway Remodeling in Asthma

In the pathogenesis of asthma symptoms, bronchospasm under the influence of external stimuli plays a key role (15). Simultaneously, the inflammation that occurs in the bronchi is responsible for the onset of symptoms. Currently, another distinguished disease component is AR, i.e., a process of reconstruction of the bronchi wall. Much research is currently focused on AR, the understanding of which will allow to search for new therapeutic possibilities of asthma (16, 17).

Chronic respiratory epithelium inflammation leads to changes in microvascularization, thickening of the airway walls and impaired airflow through the bronchi, and consequently to impaired ventilation (18). Thus, AR is a change of composition, content and distribution of cellular and molecular components in the airway wall (19). In asthma, it is associated with many structural changes–epithelial damage, subepithelial fibrosis, angiogenesis, hypertrophy and proliferation of myofibroblasts and myocytes and increased number of smooth muscle fibers in airway smooth muscle cells (ASMC), that increase airway smooth muscle (ASM) mass (20, 21). A number of inflammatory molecular factors are involved in these structural changes, either directly or *via* further induction of inflammatory reaction, namely eosinophilic (22). Starting with the local epithelium-derived factors, the key AR mediators include: PDGF (platelet-derived growth factor), TGFβ (transforming growth factor β, with particular emphasis on TGFβ_1_, among its three isoforms), FGF (fibroblast growth factor), EGF (epidermal growth factor), prostaglandin D2 (PGD2), CXCL2, CXCL3, IL-8, eotaxin, TSLP, CCL1, and other, which all promote ASMC migration (23, 24). The cytokines produced by T_h_2 (IL-4, IL-13) and T_h_17 cells (IL-17, IL-21, IL-22, TNFα) share the same effect. All of the inflammatory factors that are targeted by currently available and investigated biological therapies contribute to AR. The summary of their effects on particular components of AR is available in Table 1.

**Table 1:** Key molecular factors contributing to airway remodeling (in particular—the factors that are aimed by currently available or investigated biological therapies of asthma).

**Factor**	**Key effect(S) on airway remodeling**	**References**
IgE	1. Indirect contribution—IgE stimulates production of cytokines involved in airway remodeling (IL-4, IL-5, IL-13, TGFβ1, and other) during the late phase 2. Direct contribution—induction of ASM proliferation *in vitro*	1. (25) 2. (26)
IL-4	1. Increased synthesis of α-smooth muscle actin and collagen III 2. Induction of TGF-β release by airway epithelial cells	1. (27) 2. (28)
IL-5	1. Promotion of subepithelial and peribronchial fibrosis by eosinophil recruitment and subsequent production of TGFβ1	1. (29, 30)
IL-13	1. Induction of TGF-β release by airway epithelial cells 2. Changes in goblet cell density	1. (28, 31) 2. (32)
IL-17	1. Promotion of ASMC migration 2. Increase of matrix metaloproteinases 3. Cross-talk with TGFβ1 resulting in EMT 4. Stimulation of inactive fibrocytes maturation to fibroblast, which deposit collagen within ECM	1. (33) 2. (34) 3. (35) 4. (36)
TSLP	1. Promotion of collagen deposition 2. Goblet cells hyperplasia 3. Local eosinophil recruitment in airway 4. Increase in type-I collagen and α-SMA expression in human lung fibroblasts	1, 2, 3. (37) 4. (38)

Currently, the possible role of epithelial-mesenchymal transition (EMT) in AR is also strongly discussed. EMT is a transformation of epithelial cells into mesenchymal-like cells by loss of their epithelial traits (39). Features of EMT in AR are currently intensively studied and emerging studies confirm that EMT occurs in AR in asthma (40, 41). A major mediator of that process is TGFβ_1_, which has been proven to induce EMT of airway epithelial cells–this process occurs to a greater extent in cells of asthmatic than of non-asthmatic patients (42). It is thus worth to pay attention to the inflammation mediators which are targeted by biological therapies of severe asthma and their effect on TGFβ_1_-mediated EMT [eg. IL-4, IL-17 (35, 43)]. *In vitro* studies also show that neutrophils from severe asthmatics induce EMT in healthy bronchial epithelial cells *via* TGFβ_1_ dependencies (44). A need for further research in this area is suggested in the literature (40, 45, 46).

As a result of AR, patients may experience irreversible airway obstruction which leads to worsening of lung function, airway dilatation and response to bronchodilators. AR thus significantly contributes to the development and long-lasting persistence of asthma symptoms (16, 47–49).

## Severe Asthma and its Biological Therapy

Severe asthma affects 3.6–10.0% of patients with asthma (50–52), which corresponds to around 4 million patients globally. Currently, much research is focused on pathomechanisms of severe asthma and development of its new biological therapies (53). Although it is much less prevalent than mild and moderate asthma, severe asthma contributes to about 60% of costs associated with this disease, mainly due to drug costs (54, 55).

The ground-breaking achievement in severe asthma treatment was the introduction of its first biological treatment—anti-IgE monoclonal antibody omalizumab. The following years brought further biological agents aimed at different factors, including IL-5, IL-5R, IL-13, IL-4R, and other. Each of these drugs blocks a certain immunological pathway triggering and controlling the allergic or non-allergic airway inflammation. With the now-available monoclonal antibodies in asthma, clinicians may select a drug according to asthma phenotype. Currently, approved by the FDA and available on the market are: omalizumab, mepolizumab, benralizumab, reslizumab, and dupilumab (56).

Omalizumab is a humanized IgG1/κ monoclonal antibody that binds to the IgE immunoglobulin Fc fragment (57). Thus, it inhibits the main mediator of type I reaction pathway. By binding blood-circulating free IgE molecules, it inhibits the activation of mast cells and basophils (58). Launched in 2003, omalizumab has been used in severe allergic asthma and, since 2014, in chronic urticaria. Omalizumab is the very first monoclonal antibody included in the GINA recommendations (in 2004) on step 5. as an addition to standard therapy with high doses of inhaled steroids, β2-agonists and other drugs. Clinical and observational studies conducted over several years of using omalizumab have proven that it improves asthma control and relieves its symptoms, reduces exacerbation risk and improves lung function (59–61). Long-term safety of this drug was demonstrated in adults in terms of oncological safety and pregnancy (62–64) and in children (65).

Mepolizumab–another biological drug for severe asthma treatment—was registered in 2015. This antibody binds IL-5, which prevents it from binding to the IL-5R α subunit on eosinophils. This drug is thus used in patients with eosinophilic asthma as by blocking the IL-5 signaling, the patient's eosinophil population is reduced, which leads to clinical improvement (66). Clinical and observational studies confirmed that mepolizumab used in the treatment of severe eosinophilic asthma improves asthma control, reduces the number of exacerbations and doses of steroids used and improves lung function (67, 68). Importantly, both mepolizumab and omalizumab exhibit a comparable safety profile (69).

Whilst having achieved greatly beneficial clinical effects with biological treatment, as research in severe asthma progressed, further drugs were introduced by FDA, including: benralizumab (which targets IL-5R α subunit), dupilumab (which inhibits IL-4 and IL-13 signaling) and reslizumab (anti-IL-5 antibody) (56, 70, 71). The summary of their biological and clinical effects is available in Table 2.

**Table 2:** Summary on biologic therapies for the treatment of severe asthma and with their clinical effects and confirmed effects on AR.

	**Drug**	**Form**	**Target**	**Biological effects**	**Clinical effects**	**Effects on airway remodeling**	**Other fda-approved indications**
FDA—approved monoclonal antibodies for treatment of moderate-to-severe asthma	Omalizumab	Humanized IgG1/κ, monoclonal antibody	IgE	° ↓ circulating total IgE ° Down-regulation of FcεRI receptors on basophils, mast cells, and dendritic cells	° Improvement of lung function (FEV1) ° Improvement of quality of life (AQLQ) ° Improvement of asthma control (ACT) ° ↓ oral and inhaled corticosteroid use ° Reduction in exacerbation and hospitalization frequency (59)	° Reduction of production of TNF-α, TGFβ and IL-4 in bronchial epithelial cells (72) ° Prevention of ASM cell remodeling *in vitro*(73) ° Reduction of airway wall thickness in computed tomography (74, 75)	° Chronic idiopathic urticaria
	Mepolizumab	Humanized IgG1/κ, monoclonal antibody	IL-5	° Blockage of IL-5/IL-5R binding on eosinophils ° ↓ blood eosinophils ° ↓ sputum eosinophils	° Reduction in exacerbation frequency vs placebo ° Improvement in AQLQ vs placebo ° No significant effect on FEV1, PEF, PC_20_(76)	° Reduction of airway remodeling markers (tenascin, lumican, and procollagen III) and airway eosinophils expressing TGFβ1 in bronchial reticular basement membrane and reduction of TGFβ1 in bronchioalveolar lavage after mepolizumab treatment (77) ° Reduction of AR observed in computed tomography (78)	NA
	Benralizumab	Humanized IgG1/κ, monoclonal antibody	IL-5 Receptor alpha subunit (IL-5Rα)	° ↓ eosinophils and basophils *via* antibody dependent cell mediated cytotoxicity (ADCC)	° Reduction in exacerbation frequency ° No significant effect on FEV1 ° Mixed data on quality of life and asthma symptom scores (79)	° Decrease in airway smooth muscle mass (predicted using computational modeling approach) (80)	NA
	Dupilumab	human IgG4 monoclonal antibody	IL-4 Receptor alpha subunit (IL-4Rα)	° Blockage of IL-4/IL-4Rα binding ° Blockage of IL-13/ IL-4Rα binding	° Reduced rate of severe asthma exacerbations and improved lung function (FEV1), asthma control and quality of life (81, 82)	Studies on *in vitro* or *in vivo* effects of dupilumab on airway remodeling are currently non-available	° Eczema ° Moderate-to-severe atopic dermatitis in adolescents ° Chronic rhinosinusitis with nasal polyps
	Resliuzmab	humanized IgG4/κ mAb	IL-5	° Blockage of IL-5/IL-5R binding ° ↓ circulating eosinophils ° ↓ sputum eosinophils	° Reduced exacerbations, improved FEV1, forced vital capacity, the 7-item Asthma Control Questionnaire (83)	Studies on *in vitro* or *in vivo* effects of reslizumab on airway remodeling are currently non-available	NA
Drugs investigated (currently or previously) in severe asthma treatment	Secukinumab	human IgG1κ monoclonal antibody	IL-17A	° Blockage of IL17A -, −17F -, −17A/F heterodimer -, and −17E–(IL-25)/IL-17RA binding	NA	Studies on *in vitro* or *in vivo* effects of secukinumab on airway remodeling are currently non-available	° Plaque psoriasis ° Psoriatic arthritis ° Ankylosing spondylitis ° Discontinued in asthma
	Brodalumab	human, IgG2 monoclonal antibody	IL-17 receptor A (IL-17RA)	° Blockage of IL17A -, −17F -, −17A/F heterodimer -, and −17E–(IL-25) /IL-17RA binding	° No significant improvement in lung function (FEV1) and asthma control in subjects with inadequately controlled moderate to severe asthma (84)	Studies on *in vitro* or *in vivo* effects of brodalumab on airway remodeling are currently non-available	Plaque psoriasis
	Tralokinumab	Human IgG4 monoclonal antibody	IL-13	° Blockage of IL-13/IL-13Rα1 ° Blockage of IL-13/IL-13Rα2 binding	° Inconsistent effects on annualized asthma exacerbation rate (85)—development of tralokinumab in severe asthma was discontinued by the producer after this study (86)	° No significant effect on bronchial eosinophilic count ° No significant reduction of airway remodeling in bronchial biopsy features–Airway smooth muscle	° None available (possibly in atopic dermatitis in the future) (88) ° Discontinued in asthma
					° No significant improvement of lung function (FEV1) (87)	area, RBM thickness, collagen type IV, periostin, TGFβ and other (87)	
	Secukinumab	Humanized IgG4 monoclonal antibody	IL-13	° Blockage of IL-13/IL-13Rα1 ° Blockage of IL-13/IL-13Rα2 binding	° Decrease in asthma exacerbations incidence ° Improved lung function (FEV1%) (89)	° Greater clinical effects (decrease in exacerbation rate and improvement in lung function) in patients with high serum periostin levels – a protein contributing to airway remodeling (90)	° None available (possibly in atopic dermatitis in the future)(91) ° Discontinued in asthma
	Tezepelumab (AMG 157)	human, IgG2 monoclonal antibody	TSLP	° Blockage of TSLP/TSLP-receptor binding	° Inhibition of late allergen-induced asthmatic response (FEV1) (92) ° Reduction of annualized asthma exacerbation rate (93)	° Studies on *in vitro* or *in vivo* effects of tezepelumab on airway remodeling are currently non-available	NA

*The table includes approved and emerging therapies with published human data. NA, none available*.

## Effects of Particular Biological Drugs on Airway Remodeling

AR in asthma is mainly caused by long-term, uncontrolled airway inflammation (16). With the duration of the disease and its symptoms, structural changes in the bronchi progress, which may lead to a significant and long-term impairment of lung function (49). Considering the above and the immunomodulatory effect of biological therapies, it may be assumed that these drugs may significantly affect AR. However, data on this subject are limited and few studies cover this clinical aspect. A summary of research covering biological therapies' impact on AR is available in Table 2.

### Omalizumab

Roth et al. described the effects of IgE-contained serum from allergic asthma patients on ASM cells. The effects of such incubation were: ASM cells proliferation, deposition of type-I collagen in 48 h and of fibronectin in 24 h. A 1 h pre-incubation of ASM cells with omalizumab, prevented these three effects. The addition of allergens did not increase the IgE-dependent effects on cells incubated in omalizumab (73). Another interesting study on omalizumab was published by Huang et al. (72), in which the authors analyzed of omalizumab on allergen- and IL1β-stimulated proinflamatory cytokine and nitric oxide production in human bronchial epithelial cells (BECs) and compared them to those of budesonide. In that study omalizumab shared similar effects as budesonide in decrease of TNF-α, TGFβ and IL-4 production (72).

In 2012 Hoshino and Ohtawa compared 16 patients on omalizumab to 14 patients with conventional severe asthma treatment and measured their airway dimensions with high-resolution computed tomography (HRCT). A 16-week omalizumab treatment significantly reduced the airway wall thickness measures: airway wall area corrected for body surface area (WA/BSA)−13.7 to 12.1 mm^2^/m^2^, percentage wall area (WA%)−71.1 to 64.7% and wall thickness (T)/√BSA−1.21 to 0.92 mm/m. Luminal area (Ai/BSA) at the right apical segmental bronchus significantly increased (4.8 to 6.4 mm^2^/m^2^) and the percentage of sputum eosinophils significantly decreased. These effects were not observed in the conventional therapy group (74).

Tajiri also studied effects of omalizumab in regard to AR in a 48-week follow-up of omalizumab in 26 patients (CT measurements were analyzed in 14 patients). A significant reductions of WA% was observed (57.1 vs. baseline 62.0) and a small, but significant, increase in Ai/BSA (12.1 vs. baseline 12.0) (75).

However, Przybyszowski et al. reached slightly different results to the above. The authors analyzed changes of HRCT airway dimensions in 12 patients before and after at least 4 months of omalizumab treatment. They observed a decrease in airway wall area and WA/BSA, but no changes in WA% nor in luminal area to total bronchial area ratio (94).

### Mepolizumab

Flood-Page published in 2003 a study on the effects of mepolizumab on AR markers in bronchial biopsies of 24 atopic asthmatics from a randomized, double-bind, placebo-controlled study, which were obtained before and after three mepolizumab infusions. Compared to placebo, treatment with mepolizumab significantly reduced the expression of three extracellular matrix proteins: tenascin, lumican and procollagen III in the reticular basement membrane. Moreover, mepolizumab significantly reduced the number and percentage of airway eosinophils expressing TGFβ_1_ mRNA and decreased TGFβ_1_ in BAL fluid (77).

Only one clinical study (12-month mepolizumab vs. placebo trial, 61 subjects) analyzed the effect of mepolizumab on AR. The mean change in CT measured wall area and total area corrected for body surface area was significantly greater in treatment group than in placebo group. In fact, in mepolizumab group the values decreased whereas in placebo group an increase of these parameters was observed (78).

### Benralizumab

A very interesting approach in assessing effects of benralizumab on AR was taken by Chachi et al. (80). The researchers used bronchial biopsies of 15 patients on benralizumab and 10 patients receiving placebo, which were collected from subjects with eosinophilic asthma during a previous phase I multicenter, randomized, double-blind, placebo-controlled trial (95). The eosinophil count in airway lamina propria was assessed in pre- and post-treatment biopsies. It decreased significantly in benralizumab group by 66.4% and by 88% relative to placebo. Knowing the mechanism of action of benralizumab, and with the observed mean change in eosinophil count, the authors used a computational model to predict effects of this drug on AR. They concluded that in benralizumab group the drugs pro-apoptosis efficiency was 47%, corresponding to a consequent 29% relative reduction of ASM mass. Additionally, in the benralizumab group, a non-significant reduction in the number of tissue myofibroblasts was observed. The authors suggest that as ASM cells do not express IL-5R, the effects of benralizumab on ASM mass are an indirect effect of reduced eosinophilic inflammation. They proposed an assumption that depletion of local eosinophils results in decrease of airway TGFβ–a major growth factor contributing to AR, which is majorly expressed in lungs by the eosinophils.

### Other Antibodies (Dupilumab, Reslizumab, Secukinumab, Brodalumab, Tralokinumab, Lebrikizumab, Tezepelumab)

To authors knowledge, no study covered any aspect of AR alleviation (either *in vitro, in vivo* in animal models or *in vivo* in humans) in therapy with FDA-approved dupilumab and reslizumab. The issue was also not addressed regarding secukinumab, brodalumab, and tezepelumab—which were clinically studied in asthma, but are now discontinued in this indication.

Fragmentary data can be found regarding tralokinumab and lebrikizumab. In a phase 2 trial of tralokinumab in asthma, features of AR in bronchial biopsies (ASM area, RBM thickness, collagen type IV, periostin, TGFβ and other) were not reduced, neither was the bronchial eosinophilic infiltration (87). Lebrikizumab in turn showed greater clinical effects (decreased exacerbation rate and improved lung function) in asthmatic patients with high serum periostin levels (protein contributing to AR) in a phase 2 trial (90). However, the two drugs' development in asthma is also discontinued.

## Summary

According to the American Thoracic Society (ATS) 2017 expertise—and other researchers' opinions (16, 96)–it should be further investigated whether, and if so, to what extent, biological therapy of asthma significantly affects AR and what are the clinical consequences of such an effect. Moreover, research on AR was indicated by ATS as crucial in the development of knowledge about asthma and its treatment. The need to monitor AR in future clinical studies is pointed out as an important aspect of response to modern biological treatment of severe asthma. These recommendations also highlight the need to study the impact of currently available biological preparations on AR and to look for new drugs that alleviate it (97).

The development of biological therapies has opened a new chapter in treatment of severe asthma. Since the introduction of the first monoclonal antibody in this disease—the anti-IgE drug omalizumab in 2004—a new range of biology-oriented therapy emerged. With the arrival of the subsequent antibodies targeting other molecules involved in asthma pathophysiology, it became possible to treat the most severely ill patients using phenotype-oriented drugs. However, as only since recent years new drugs in this field arrive, little is known about their effect on the AR—a key clinical feature of severe asthma and its long-lasting consequences. The data available to date confirms with a high degree of probability only the beneficial role of omalizumab in reversing AR. Some promising studies cover this topic in regard to mepolizumab and benralizumab. Yet, future research of available and upcoming biological therapies in severe asthma shall address this clinical issue as an important feature of long-term severe asthma management.

## Author Contributions

GK and MP created the concept of the paper. GK conducted the literature research and wrote the manuscript. PK and MP revised the paper.

## Conflict of Interest

The authors declare that the research was conducted in the absence of any commercial or financial relationships that could be construed as a potential conflict of interest.
